# Supplementary material for: Increased awareness and decreased acceptance of genome-editing technology: The impact of the Chinese twin babies
Source: PLoS One. 2020 Sep 18;15(9):e0238128. doi: 10.1371/journal.pone.0238128 (PMC7500613; doi:10.1371/journal.pone.0238128)
Supplement: S1 Text — (DOCX) [file pone.0238128.s001.docx]

**Questionnaire**

**Food Questions (2018, 2019)**

or

**Survey Related to Science and Technology (2016)**

To the registered monitor of MACROMILL, INC.,

We ask that you thoroughly adhere to the "Confidential Policy" in the monitor agreement. Please never reveal the information you acquired from this survey to anyone or any third party (this includes refraining from posting on the internet or social networking services [SNS]).

……………………………………………………………………………………

**(2018, 2019 only)**

**Q1.** How important are each of the following social problems in Japan?

Answer each based on the following scale:

1: Not at all

2: Not important

3: Cannot decide

4: Important

5: Very important

1．Fishery, agriculture, and food production

2．Impact of climate change on agriculture and fishery production

3．Declining birthrate and support for child bearing

4．Disaster prevention

5．Aging population and support for senior citizens

6．Food safety and food security

7．CO2 and climate change

8．Rural development in Japan

9．Depopulation in rural Japan

10．Immigration

11．Social security problems

12．Diplomatic problems

……………………………………………………………………………………

**(2018, 2019 only)**

**Q2.** Do you think we have to promote the following technological developments in Japan? Per your opinion, please choose the most suitable response.

Answer each based on the following scale:

1: Do not promote

2: Should not promote

3: Cannot decide

4: Should promote

5: Promote

1．Earthquake resistance and disaster prevention

2．Biotechnology in food production (i.e., genome editing or genetic engineering)

3．Regenerative organ medicine

4．Robotic technology

5．Climate change simulations

6．Driver safety

7．AI technology applications

8．Less nature-dependent technology for fishery production

9．Technology takeover

10．Medical applications for genetic information

11．Space development

12．Ocean development

……………………………………………………………………………………

Please read the following sentences:

Genome editing technology can remove or replace target genes. Unlike conventional genetic modification technology, foreign genes do not remain in DNA. Previous research reveals that similar mutations are occurring in the natural environment. Currently, genome editing research is utilized for agricultural and fishery product breeding purposes and for medical applications.

**Q3.** How much did you know about genome editing technology?

1. Nothing at all

2. Not much

3. Have heard of it

4. Know some information

5. Very knowledgeable

……………………………………………………………………………………

If the respondent chose 3, 4, or 5 in Q3, continue to Q4.

If the respondent chose 1 or 2 in Q3, skip to Q7.

……………………………………………………………………………………

**Q4.** Please choose the most suitable description of your genome editing technology knowledge gained from media coverage:

1. Explanation of the technology itself

2. Application to fishery or agricultural breeding technology

3. Explanation of the risks of technology adoption

4. Medical applications of the technology

5. Application to fertilized human eggs (2018, 2019 only)

6. Others

7. Do not remember (skip to Q7)

……………………………………………………………………………………

**Q5.** Please choose the top three answers that most closely match your impression when you first heard the words, or explanation of, genome editing technology (2016).

Please evaluate each of the impressions on a following four-point Likert scale (2018, 2019).

Answer each based on the following scale: (2018, 2019)

1. Strongly disagree

2. Disagree

3. Somewhat agree

4. Strongly agree

1. Interesting (or impressive)

2. Amazed by this technological advancement

3. Looking forward to its adoption

4. Worried about its adoption

5. Concerned about its side effects or unknown effects

6. Concerned about ethical issues (2018, 2019 only)

7. Do not understand the reason for its adoption

8. Complicated explanation

9. Other

……………………………………………………………………………………

**Q6.** In the process of developing a new variety, such as a high-yield variety or a high-quality rice, breeders have employed various breeding technologies. How much do you know about each of the following breeding technologies? Answer each based on the following scale:

1: Nothing at all

2: Not much

3: Have heard of it

4: Know some information

5: Very knowledgeable

1. Adoption research

2. Pure line selection

3. Cross breeding

4. Mutation breeding

5. Accelerating generation

6. DNA markers

7. Genetic modification

8. Genome analysis

……………………………………………………………………………………

**Q7.** How much biology did you study in school? Please choose the best response based on your experience. Base your answer on your highest level of study.

1. None

2. High school level

3. University level

4. Postgraduate level

……………………………………………………………………………………

**Q8.** What is your highest level of education?

*If you are currently enrolled in school, answer based on your anticipated level of education.

1. Junior high school graduate

2. High school graduate

3. Junior college or vocational or special school graduate

4. Dropped out of university (Some university classes)

5. University or college graduate

6. Graduate school graduate

7. Others

……………………………………………………………………………………

**食品（技術）に関するアンケート**

*2016年は技術、2018年、2019年は食品

下記アンケートにご協力お願いいたします。

-------------------------------------------------------------------------------

**当アンケートの回答者の皆様へお願い**

-------------------------------------------------------------------------------

マクロミルモニタの皆様にはモニタ規約にて「調査についての守秘義務」の徹底をお願いしています。

当アンケートの内容および当アンケートで知り得た情報については、決して第三者に口外しないよう（掲示板やホームページへの書き込みを含む）、ご協力お願いします。

……………………………………………………………………………………

(2018, 2019 のみ設定)

**Q1 現在、日本が直面する様々な課題について、それぞれどの程度重要だと思いますか。**

**あなたの考えに近いものをお選びください。**

1．全く重要でない 2．どちらかというと重要でない

3．どちらともいえない　　　　　 4．どちらかというと重要である

5．とても重要である

| 1 | 漁業や農業など食料生産 |  |
| --- | --- | --- |
| 2 | 気候変動による農水産物生産への影響 |  |
| 3 | 少子化対策・子育て支援 |  |
| 4 | 防災・被災地支援 |  |
| 5 | 高齢化対策・高齢者支援 |  |
| 6 | 食の安全・安心 |  |
| 7 | CO2排出削減や気候変動 |  |
| 8 | 地方創生の推進 |  |
| 9 | 過疎化対策 |  |
| 10 | 難民・移民問題 |  |
| 11 | 社会保障制度の問題 |  |
| 12 | 外交問題 |  |

……………………………………………………………………………………

(2018, 2019 のみ設定)

**Q2 日本の科学技術開発についてお聞きします。**

**以下の科学技術について、それぞれどの程度推進すべきだと思いますか。**

**あなたの考えに最も近いものをお選びください。**

1．推進すべきでない 2．どちらかといえば推進しないほうがよい

3．どちらともいえない 4．どちらかといえば推進したほうがよい

5．ぜひ推進すべきである

| 1 | 免震・耐震を含む災害対策 |
| --- | --- |
| 2 | 食料生産に利用するバイオ技術（遺伝子工学、ゲノム編集など） |
| 3 | ケガや病気に冒された臓器などを再生する再生医療（iPS細胞など) |
| 4 | 人の代わりに作業する産業ロボットの開発 |
| 5 | 気象予測などのシミュレーション技術 |
| 6 | 車の安全運転技術（高齢ドライバー支援含む） |
| 7 | AI（人工知能）技術の利用拡大 |
| 8 | 天然資源に依存しない持続可能な養殖や水産技術の開発 |
| 9 | ものづくりの継承 |
| 10 | 遺伝子治療 |
| 11 | 宇宙開発 |
| 12 | 海洋資源開発 |

……………………………………………………………………………………

**以下の説明文をお読みいただき、設問にお答えください。**

| ゲノム編集技術とは、ゲノム*を構成するDNAの狙った部分を取り除いたり、置き換えたりできる最先端の技術です。従来の遺伝子組換え技術と異なり、外部から導入した遺伝子はゲノムに残りません。また、これまでの研究から、ゲノム編集でDNAを取り除いたり、DNAを置き換えたりすることと同じ現象が、自然界でも頻繁に起こっていることがわかっています（変異（または突然変異）と呼ばれています）。現在、農水産物の品種改良や医療用途での応用を見据えて、ゲノム編集に関する研究が進んでいます。  *ゲノム：ある生物がもっている遺伝情報の全体 |
| --- |

**Q3 あなたは、「ゲノム編集技術」についてどの程度ご存知でしたか。**

1．全く知らなかった 2．ほとんど知らなかった 　3．聞いたことがあった

4．大体知っていた 5．よく知っていた

---------------------------------------------------------------------

【分岐】

回答が３，４，５の場合→Q4、Q5、Q6、…を回答

回答が１，２の場合→Q7へ

……………………………………………………………………………………

**Q4（Q3で3,4,5と回答した人のみ）**

**「ゲノム編集技術」という言葉や説明を3.「聞いたことがあった」、4.「大体知っていた」、5.「よく知っていた」と回答した方に伺います。その際、最も印象に残った言葉や説明は、どのような内容でしたか。最も当てはまるものを１つ、お選び下さい。**

1．技術を説明したもの

2．水産物や農作物、家畜の品種改良について説明したもの

3．技術利用の危険性を説明したもの

4．医療への応用事例を説明したもの

5．ヒト受精卵への応用に関する内容(2018, 2019のみ)

6．その他

７．覚えていない(Q7へ)

……………………………………………………………………………………

**Q5**

**(2016) 最も印象に残ったゲノム編集の説明を聞いたときの気持ちについて、お気持ちに近い順に１～３位までお選びください。**

**(2018, 2019) ゲノム編集に関する言葉や説明を聞いたときのあなたの印象について、それぞれ1つずつお選びください。**

**＊「ゲノム編集技術」という言葉を1.「全く知らなかった」または2.「ほとんど知らなかった」と回答した方は、前問の説明文を読んだ際の「ゲノム編集技術」に対する印象をお答えください。**

1．全く当てはまらない 2．あまり当てはまらない

3．やや当てはまる 4．とても当てはまる

| 1 | 興味深い |  |
| --- | --- | --- |
| 2 | 技術の進歩に驚いた |  |
| 3 | 今後の可能性が楽しみになった |  |
| 4 | やや心配を覚えた |  |
| 5 | 副作用や未知の影響が気になった |  |
| 6 | 倫理面での課題が気になった(2018, 2019のみ) |  |
| 7 | 技術を使用する意義がわからなかった |  |
| 8 | 説明がわかりにくかった |  |
| 9 | その他 ( ) |  |

……………………………………………………………………………………

**Q6 品種改良によって、同じ農産物（例えばお米）でも、味や収量性などが異なる多くの種類が作り出されてきました。その際、様々な品種改良技術が使われてきました。**

**以下の品種改良・遺伝資源に関する用語や事柄について、あなたはどの程度知っていましたか。**

1．全く知らない 2．ほとんど知らない 3．聞いたことがある

4．大体知っている 5．よく知っている

| 1 | 導入育種　(ほかの地域から種子を持ってきて栽培すること) |  |
| --- | --- | --- |
| 2 | 純系淘汰　(優良なものを選び出し、種子を増やしていく育種方法) |  |
| 3 | 交配育種　(異なる品種の花粉(おしべとめしべ)を合わせる技術) |  |
| 4 | 突然変異　(放射線照射などにより人為的に突然変異を起こす育種方法) |  |
| 5 | 世代促進　(温室を使って年に数回、花を咲かせる方法) |  |
| 6 | DNAマーカー選抜育種　(DNAの情報をもとに選抜する方法) |  |
| 7 | 遺伝子組換え　(外来の遺伝子を導入する育種方法) |  |
| 8 | ゲノム解析　(稲など、全遺伝子のDNA配列を解読して育種に利用すること) |  |

……………………………………………………………………………………

**Q7 あなたは生物学を勉強した経験がありますか。以下の中から最もあてはまるものをひとつお選びください。**

**＊勉強した最も高いレベルについてお答えください。**

**＊ここでいう「生物学」とは、高校で学習するレベル以上のものを指します。**

1．特になし 2．高校で学習する生物学

3．大学レベルの生物学 4．それ以上

……………………………………………………………………………………

**Q8 あなたの最終学歴をお選びください。**

**＊在学中の方は、卒業見込みとして当てはまるものをお選びください。**

1．中学卒業 2．高校卒業

3．短大・専門学校卒業 4．大学中退

5．大学卒業　　　　　　　　　　　　　　　　6．大学院修了

7．その他

……………………………………………………………………………………
