# Supplementary material for: Increased awareness and decreased acceptance of genome-editing technology: The impact of the Chinese twin babies
Source: PLoS One. 2020 Sep 18;15(9):e0238128. doi: 10.1371/journal.pone.0238128 (PMC7500613; doi:10.1371/journal.pone.0238128)
Supplement: S2 Table — (DOCX) [file pone.0238128.s003.docx]

**S2 Table. Awarenesses and their corresponding impressions**

|  | “Very knowledgeable” “Know” or “Have heard” (%) | | | Positive (%) | | | Negative (%) | | | Neutral (%) | | |
| --- | --- | --- | --- | --- | --- | --- | --- | --- | --- | --- | --- | --- |
|  | 2016 | 2018 | 2019 | 2016 | 2018 | 2019 | 2016 | 2018 | 2019 | 2016 | 2018 | 2019 |
| All | 28.8 | 41.4 | 51.8 | 46.2 | 37.7 | 27.6 | 36.8 | 29.1 | 35.8 | 17.0 | 33.2 | 36.6 |
| Male | 35.5 | 54.0 | 64.6 | 49.4 | 45.7 | 32.5 | 34.9 | 23.2 | 29.2 | 15.7 | 31.1 | 38.3 |
| Female | 22.1 | 28.7 | 39.0 | 40.6 | 21.8 | 19.3 | 40.2 | 40.8 | 47.0 | 19.3 | 37.3 | 33.7 |
| Male 20s | 33.5 | 50.8 | 54.8 | 52.3 | 46.2 | 44.4 | 33.0 | 19.2 | 22.2 | 14.8 | 34.6 | 33.3 |
| Male 30s | 33.2 | 53.2 | 57.4 | 47.1 | 45.1 | 29.2 | 31.8 | 21.6 | 33.3 | 21.2 | 33.3 | 37.5 |
| Male 40s | 29.0 | 53.2 | 59.1 | 52.2 | 52.8 | 36.4 | 34.8 | 18.9 | 27.3 | 13.0 | 28.3 | 36.4 |
| Male 50s | 36.8 | 55.6 | 74.2 | 54.7 | 40.4 | 25.7 | 32.6 | 35.1 | 27.7 | 12.8 | 24.6 | 46.5 |
| Male 60s | 44.8 | 57.3 | 77.8 | 42.9 | 44.8 | 30.2 | 41.0 | 20.9 | 34.0 | 16.2 | 34.3 | 35.9 |
| Female 20s | 27.4 | 28.2 | 38.7 | 47.5 | 40.7 | 23.3 | 31.2 | 25.9 | 46.5 | 21.3 | 33.3 | 30.2 |
| Female 30s | 20.3 | 21.0 | 33.8 | 32.6 | 21.7 | 18.2 | 50.0 | 47.8 | 34.1 | 17.4 | 30.4 | 47.7 |
| Female 40s | 18.7 | 27.4 | 31.0 | 50.0 | 17.4 | 18.4 | 29.5 | 56.5 | 42.1 | 20.5 | 26.1 | 39.5 |
| Female 50s | 20.0 | 32.3 | 42.2 | 25.0 | 11.8 | 22.0 | 52.3 | 52.9 | 47.5 | 22.7 | 35.3 | 30.5 |
| Female 60s | 23.9 | 34.7 | 49.7 | 44.4 | 20.0 | 15.4 | 40.7 | 25.7 | 58.5 | 14.8 | 54.3 | 26.2 |

Source: Survey results
