# Supplementary material for: Increased awareness and decreased acceptance of genome-editing technology: The impact of the Chinese twin babies
Source: PLoS One. 2020 Sep 18;15(9):e0238128. doi: 10.1371/journal.pone.0238128 (PMC7500613; doi:10.1371/journal.pone.0238128)
Supplement: Attachment — Submitted filename: 3-R3. Reply to reviewer comments.docx [file pone.0238128.s007.docx]

Reply to Comments from Reviewer #3.

**Comment #1-2, 4:**

#1 All the text needs a language revision in order to improve some typos and grammatical errors.

#2 Please, check in all the text the use of references as subject. This is not correct, use the form Surname et al. [reference] verb …

#4 I would suggest checking the authors guidelines for the manuscript format.

**Reply**

Thank you for your comments. We have used an English editor to thoroughly check for such errors and have changed the format in accordance with *Plos One* requirements.

**Comment #3:**

I would suggest checking the use of abbreviation in the abstract and in the main text, they need to be reported in the extended form at the first use.

**Reply**

Thank you for your comment. We now have checked the manuscript thoroughly to ensure it follows the *Plos One* requirement.

**Comment #5 - #8:**

#5: I would suggest providing more information about the target population included in the Macromill database, the geographic area, and how the list of the company has been built. Is it actually representative of the entire population? All these details are required to understand the possible biases and limitations of the survey which need to be better discussed.

#6: I would suggest providing data about non responders and discuss the possible role of this proportion of subjects on the study results. This can be a source of bias and limitations. Are more characteristics of the investigated population available, such age, education, or other? The same about who did not respond.

\# 7: I would suggest providing more detail about “how” the survey was conducted. The modality, if interviewers were adopted, which language, if the survey was validated and the process of validation. In general, more details about the methods are required to understand the actual value of provided results and specifically the inference from these results.

#8: I would suggest adding a methods section with deep detailed description of survey development with validation, survey distribution, target population (geographic area, language, details about list). The Authors should add this section with the aim to allow the repetition of the study by other investigators, key information and steps used to product the results need to be reported.

**Reply**

Thank you for these suggestions in #5 - #8. Since they relate to each other, our reply groups them below, as organized by the ways in which we have responded to them.

1. Pre-registered sample

(1) How it was created

In the Survey Description sub-part of Survey and Results, we now describe in detail how the pre-registered sample was created. The number of respondents in the pre-registered sample is indicated in Table 1, and the summary statistics in S1 Table.

(2) Summary statistics and representativeness of our survey

In order to show the representativeness of our surveyed sample, S1 Table includes summary statistics of the pre-registered sample and the respondents to our survey, which are further divided in Tables S2 - S3 into respondents “Being aware” and “Not being aware” about genome editing technology. Although we are unable to check for the statistical significance of differences, the pre-registered and surveyed samples are quite similar to one another in occupation and location.

We excluded from further regression analysis respondents who were unaware of the technology. In other words, our results exclude respondents who have no information, and this may enhance the supportiveness of the mean attitude. However, including them also results in drawbacks if we provide them additional information to answer the questions since their attitude may depend on what information we provide. We explain this possible exclusion bias in the survey section of the paper.

2. Survey method and summary statistics of our survey

In the Survey description section, we explain how the respondents were solicited. Respondents received announcement from the survey company and replied to that online survey questionnaire if they wished to answer. The survey opened until the number of respondents reached the requested number of participants. Respondents were incentivized to complete the survey by receiving a small gift or points that could be used for online shopping.

As explained above, summary statistics are shown in S1 Table, and the survey questionnaire in both the original language (Japanese), and English translation are presented in S1 Text. Since respondents may judge whether or not to participate into the survey based on the questionnaire title, we did not use the words “genome editing technology” in the title, in order to avoid extreme opinions.

3. Repetition of the result

We provide summary statistics for both pre-registered and surveyed samples. Survey samples, especially those used for the regression analysis, are provided in dx.doi.org/10.17504/protocols.io.bhxuj7nw. A survey questionnaire is also provided in S1 Text. It should be possible for others to replicate the results given these observations and commands.

Thank you again for your comments.
